# Anthelmintic Effect of Biocompatible Zinc Oxide Nanoparticles (ZnO NPs) on *Gigantocotyle explanatum*, a Neglected Parasite of Indian Water Buffalo

**DOI:** 10.1371/journal.pone.0133086

**Published:** 2015-07-15

**Authors:** Yasir Akhtar Khan, Braj Raj Singh, Rizwan Ullah, Mohd Shoeb, Alim H. Naqvi, Syed M. A. Abidi

**Affiliations:** 1 Section of Parasitology, Department of Zoology, Aligarh Muslim University, Aligarh, 202002, India; 2 Centre of Excellence in Material Science (Nanomaterial), Department of Applied Physics, ZHCET, Aligarh Muslim University, Aligarh, 202002, India; Institute for Materials Science, GERMANY

## Abstract

Helminth parasites of veterinary importance cause huge revenue losses to agrarian economy worldwide. With the emergence of drug resistance against the current formulations, there is a need to focus on the alternative approaches in order to control this menace. In the present study, biocompatible zinc oxide nanoparticles (ZnO NPs) were used to see their *in vitro* effect on the biliary amphistomes, *Gigantocotyle explanatum*, infecting *Bubalus bubalis* because these nanoparticles are involved in generation of free radicals that induce oxidative stress, resulting in disruption of cellular machinery. The ZnO NPs were synthesized by using egg albumin as a biotemplate and subsequently characterized by Scanning Electron Microscopy (SEM), Transmission Electron Microscopy (TEM), X-ray Diffraction and Spectrophotometrical, which showed that ZnO NPs were highly purified wurtzite type polycrystals, with a mean size of 16.7 nm. When the parasites were treated with lower concentrations (0.004% and 0.008%) of the ZnO NPs, the worms mounted a protective response by stimulating the antioxidant system but the treatment of *G*. *explanatum* with 0.012% ZnO NPs produced significant inhibition of the antioxidant enzymes like superoxide dismutase (SOD) (p< 0.05) and glutathione S- transferase (GST) (p<0.01), while the level of malondialdehyde (MDA), a lipid peroxidation marker, was significantly (p< 0.01) elevated. SEM and histopathology revealed pronounced tegumental damage showing the disruption of surface papillae and the annulations, particularly in the posterior region near acetabulum. The under expression of a number of polypeptides, loss of worm motility in a time dependent manner, further reflect strong anthelmintic potential of ZnO NPs. It can be concluded that the anthelmintic effect might be due to the production of reactive oxygen species that target a variety of macromolecules such as nucleic acid, protein and lipids which are involved in different cellular processes.

## Introduction

Helminth infections are widespread throughout the world, ranging from tropical, subtropical to temperate climates, affecting both humans as well as livestock animals and cause huge economic losses worldwide. Only *Fasciola* sp. (*Fasciola gigantica* and *Fasciola hepatica*) causes worldwide economic losses to the tune of 3.0 billion USD per annum in terms of mortality and decrease in production of milk, wool and meat yield [1]. *Gigantocotyle explanataum* is another neglected amphistome parasite infecting the bile duct of water buffalo. The prevalence of infection is very high in Indian subcontinent. The personal survey at the local abattoirs reveals that about 60% buffaloes sacrificed had *G*. *explanatum* infection. The worldwide economic loss due to *G*. *expalanatum* cannot be well said because of non-availability of data, but a recent report (Anon, 2011–2012) revealed its potential threat in India [2]. *G*. *explanatum* infected livers show connective tissue proliferation, hemorrhages at the site of attachment, hypertrophy and hyperplasia in the bile duct, thereby seriously affecting the health and productivity of the infected animals [3].

In the absence of an effective vaccine, chemotherapy is the main effective tool to combat and control helminth parasites, but there are several reports of emergence of anthelmintic resistance in parasites [4–6]. Therefore, the development of effective alternative is important, that can be achieved by nanoparticle based drug formulations. It has been investigated in case of bacteria that the drug toxicity resulted from the use of multiple antibiotics to treat drug resistant strains has led to the intervention of metal oxide nano particles which cause disruption of the morphology, membrane permeability and the transport processes [7, 8]. Among the parasites, anti-protozoan activity of the metal oxide nanoparticles has also been investigated, but no data is available in case of amphistoms. Allahverdiye et al. (2011) reviewed *in vitro* anti-leishmanial activity of TiO_2_ and AgO_2_ nanoparticles, which significantly inhibited the proliferation of parasites [9]. Different forms of NPs have been used to control number of parasites [10–12]. Zinc oxide nanoparticles (ZnO NPs) are non toxic to human beings and zinc is a necessary element for human health, but toxic to the microorganisms [13]. Nano size of ZnO and other metal derived particles gives a higher surface exposure of the atoms on nano particles and thereby exhibit different physical [14–17], chemical [16, 18–20] and a very high level of biological response [18, 21–34]. Furthermore, human cells also have good biocompatibility with ZnO nanoparticles [13] and the ZnO has been documented as a safe material by FDA, USA [35]. Previous research on the effect of ZnO NPs on bacteria has shown an increase in the level of reactive oxygen species, membrane damage and quenching of nanoparticles [13]. The antimicrobial effect of ZnO NPs is present in its electronic band gap that has been considered to be driving force for generation of reactive oxygen species [36, 37], which is the triggering agent for the intracellular disruption of homeostasis. ZnO NPs also know to inhibit biofilm formation, an evasive mechanism of bacteria against anti bacterial drugs [31]. In recent years the role of different fabricated NPs extended beyond antibacterial effect to the treatment of deadly diseases such as cancer and viral infections [24, 26–30, 33, 34]. The structural alteration in the ZnO NPs has also been used to lower the toxicity against mammalian cells [32]. Interestingly, in case of helminth parasites an effective cytochrome P450 and thioredoxin system, SOD (super oxide dismutase) and GST (glutathione-S-transferase) enzymes are present to protect themselves from the host generated oxidative stress. It is also known that free radical ions produced due to drug treatment are processed by the helminth anti-oxidant system [38]. Therefore, suppression or disruption of this protective mechanism of the parasites would be an effective target that may help in the elimination of the infection.

In last decade various methods for synthesis of nanostructure ZnO have been used [14, 19, 39, 40]. In the present study, we have synthesized ZnO NPs by using egg albumin as a biotemplate for their anthelmintic effect on *G*. *explanatum*, infecting the Indian water buffalo. The different properties of ZnO NPs have been analyzed. These biocompatible ZnO NPs have already been used as anticandidal agent [37]. Following *in vitro* treatment of *G*. *explanatum* with ZnO NPs, the key antioxidant enzymes SOD, GST and oxidative damage marker for lipid peroxidation process, malondialdehyde (MDA), polypeptide profile, topographical features of the body surface and the histopathology have been investigated.

## Materials and Methods

### ZnO Nanoparticle based studies

#### Materials

Zinc acetate, potassium bromide, ammonia and other chemicals were procured from local suppliers. All other chemicals used were of the highest purity grade available from commercial sources like Fisher Scientific (U.S.A.), Sigma (U.S.A.) etc.

#### Synthesis of ZnO NPs using egg albumin as a biotemplate

The synthesis of ZnO NPs using egg albumin as a biotemplate (denoted as “ZnO NPs”) was performed according to the previously reported method [37]. In brief, freshly extracted 30 ml egg albumin (5 mg/mL) was mixed drop-wise into 70 ml aqueous 0.25M zinc acetate [Zn(CH_3_COOH)_2_.2H_2_O] solution. The mixture was stirred 20 min at room temperature and precipitated by the addition of the ammonia (NH_3_) at ~pH 7.0 and centrifuged at 5000 rpm for 10 min. The obtained pellet was twice washed carefully with sterile distilled water and dried in the vacuum oven. The obtained dried powder of ZnO NPs was subjected to sintering at 400°C for 3 hours and stored until used.

#### Characterization of ZnO NPs

The synthesis of ZnO NPs at the preliminary level was confirmed by recording the absorbance (A) on a UV-vis spectrophotometer (Perkin Elmer Life and Analytical Sciences, CT, USA) in the wavelength range of A_200_ to A_800 nm_ [37]. Further, authentication of the successful synthesis of ZnO NPs *was done by* X-ray diffraction (XRD) pattern. For that, the ZnO NPs powdered sample was recorded on MiniFlex II benchtop XRD system (Rigaku Corporation, Tokyo, Japan) operating at 40 kV [40]. The surface morphology of ZnO NPs was determined using a JEOL- JSM-6510LV SEM machine (Tokyo, Japan) operated at a voltage of 10 kV. The transmission electron microscopy (TEM) of ZnO NPs was carried out on JEOL 100/120 kV TEM (JEOL, Tokyo, Japan) with an accelerating voltage of 200 kV. The lattice fringes and diffraction ring patterns (SAED) were examined in its high resolution (HRTEM) mode. FTIR spectra of ZnO NPs were obtained in the range 4,000 to 400 cm^-1^ with a PerkinElmer FTIR spectrophotometer by potassium bromide (KBr) pellet method [37, 40]. Spectroscopic grade KBr was used in the ratio of 1:100 and spectrum was recorded in the diffuse reflectance mode at a resolution of 4 cm^-1^ in KBr pellets.

### Anthelmintic effect of ZnO NPs

#### Preparation of ZnO nanoparticles suspension

Stock suspension of the ZnO NPs was prepared in phosphate buffered saline, pH-7.4, while the working solutions of 0.004%, 0.008%, 0.012% (w/v) were prepared with complete RPMI 1640 medium supplemented with 5% fetal bovine serum (FBS) (Himedia) and 0.5% antibiotic-antimycotic solution (Himedia).

#### Collection of Parasites

Adult parasites were collected from the bile duct of freshly slaughtered buffaloes at a municipal abattoir of Aligarh, India. Parasites were washed with Hanks’ balanced salt solution (HBSS) and used for anthelmintic studies.

#### Incubation of Parasites with ZnO NPs Suspension

A total of 20 parasites in each concentration of ZnO NPs mentioned above were incubated at 37°C for 24 hours in 50 ml of RPMI 1640 along with the controls which were devoid of NPs. After incubation, parasites were washed with PBS several times to remove the adherent particles and used for various studies. For biochemical studies, few worms were homogenized (10% w/v) to prepare cell free extract in 50 mM Tris-HCl, pH 8.0, centrifuged at 10000×g for 30 minutes at 4°C and supernatant was stored at -80°C until used. Protein content of homogenate was determined according to the dye binding method [41].

#### Parasite Motility determination

The motility of worms was periodically recorded at every 2 hour intervals up to 12 hours and finally at the 24^th^ hour of incubation in different concentrations of the ZnO NPs along with controls.

#### Super Oxide Dismutase (SOD) activity assay

The SOD level after different treatment of parasites was measured according to the method of Marklund and Marklund (1974) [42]. The specific activity of SOD has been expressed as unit/mg protein. Percent inhibition of SOD specific activity was calculated by formula: S_0_ - S_1_ / S_0_ x 100, where S_0_ = activity of control, S_1_ = activity of sample.

#### Glutathione-S-transferase (GST) activity assay

The conjugation of the GST with reduced glutathione was measured by the method of Habig et al. (1974) [43]. The specific activity of GST was calculated in terms of Unit/mg protein. The percent inhibition of GST activity was calculated by the formula: G_0_ - G_1_ / G_0_ x 100, where G_0_ = activity of control, G_1_ = activity of sample.

#### Malondialdehyde (MDA) assay for lipid peroxidation

The level of MDA, a marker for lipid peroxidation process was determined by the procedure of Buege and Aust (1978) [44]. The level was expressed in nmoles of MDA formed per ml serum sample by using molar extinction co-efficient of 1.56×10^-5^M^-1^ cm^-1^ for MDA-TBA (malondialdehude-tribarbituric acid) colored complex.

#### Sodium dodecyl sulphate polyacrylamide gel electrophoresis (SDS PAGE)

The parasite somatic proteins were precipitated with TCA/Acetone (10% w/v) in 1:1 ratio over night at -20°C and then centrifuged at 10,000×g for 30 minutes at 4°C. The supernatant was discarded and the pellet was washed four times with acetone and protein content was estimated by the dye binding method [41]. The SDS PAGE was carried out according to the method of Laemmli (1970) [45], using a 12% separating and a 5% stacking gel. The electrophoresis was carried out at a constant voltage of 100V on a Bio-Rad Mini-Protean Tetra System. Thereafter, the gel was stained with 0.25% Coomassie Brilliant Blue-R250 (CBBR- 250) dye prepared in methanol, water, acetic acid (50:40:10) solution. The image was taken on Gel Doc XR Plus system (Bio-Rad, U.S.A.).

#### Scanning Electron Microscopy (SEM) of *G*. *explanatum*

The ultrastructural changes in the metabolically active body surface, i.e. the tegument, of worms were investigated by SEM following treatment of worms with ZnO NPs. The parasites treated with 0.012% ZnO NPs were gently washed four times with PBS, pH-7.4, to remove adherent NPs and then fixed in 2.5% glutaraldehyde / paraformaldehyde solution for 5 hours. Further processing was carried out at the University Sophisticated Instrumentation Facility, AMU, Aligarh, following the standard protocol described by Tansatit et al. (2012) [46] and the specimens were observed on a JSM 6510 LV, JEOL scanning electron microscope (Tokyo, Japan) operating at 15kV.

#### Histopathology

Parasites treated with 0.012% NPs were fixed in 10% neutral buffered formalin overnight, then washed with PBS, pH-7.4 and processed for the histology. Sections at 5μm thickness were cut on a rotary microtome (Yorco, India), stained in haematoxyline and eosin and observed under bright field on Axioscope A1 microscope (Zeiss, Germany), fitted with a digital camera. The representative photographs were taken to record the extent of damage.

### Statistical Analysis

The data was subjected to statistical analysis on One Way ANOVA by using Tukie’s Comparison Test on statistical software- ‘GraphPad Prism 5’ and level of significance was determined. Data is presented as Mean±SE and p< 0.05 was considered as significant.

## Results

### Properties of ZnO NPs

**Fig 1:**
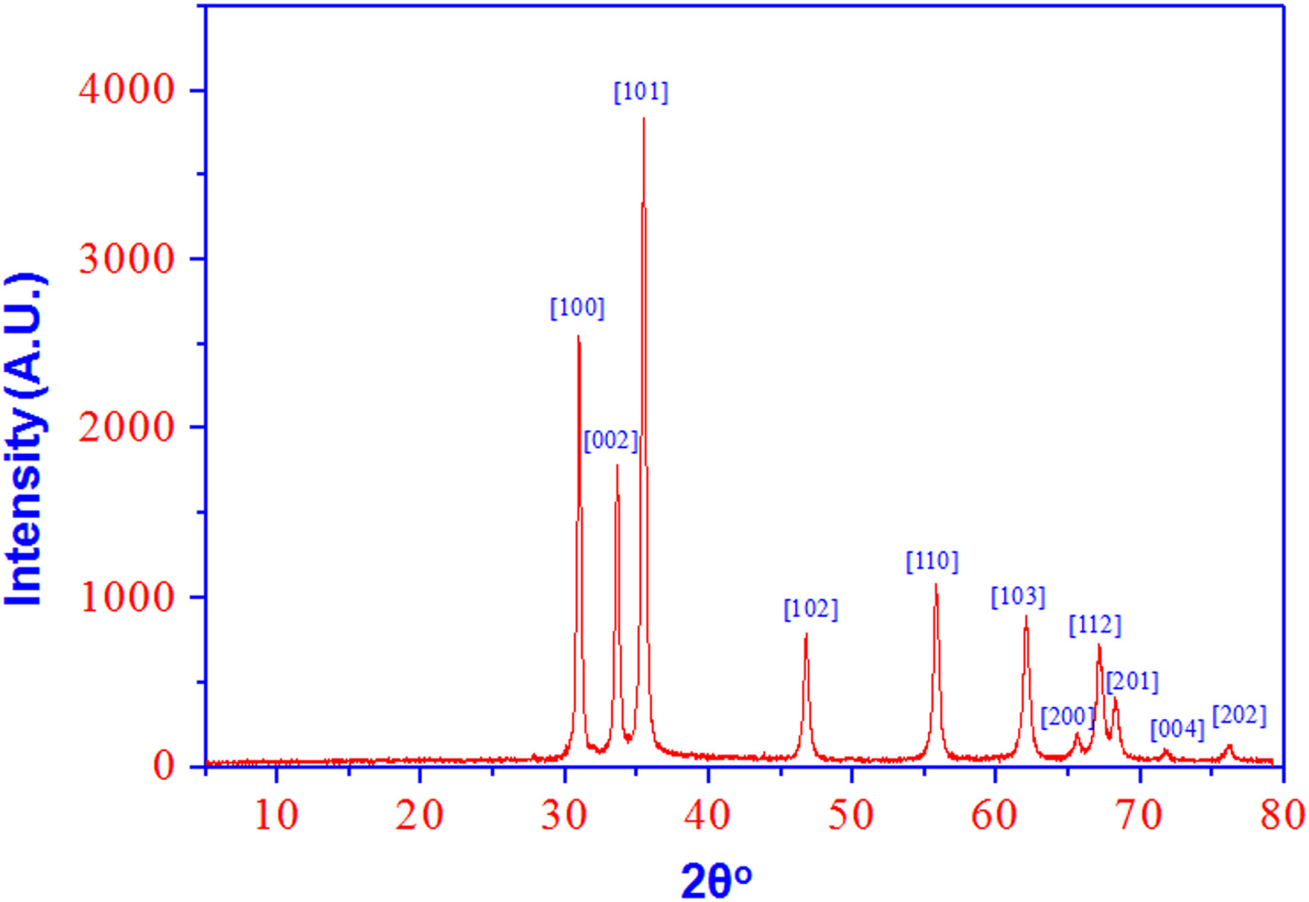
XRD pattern of ZnO NPs shows a typical wurtzite type polycrystals.

Initially, synthesis of ZnO NPs using egg albumin was confirmed by the X-ray diffraction (XRD) technique with Cu Kα radiation (λ = 0.15418 nm). Fig 1A shows a typical XRD pattern of ZnO NPs in the range of angle (2Ө)20–80°, which shows Bragg reflections with 2 θ values of 31.11°, 34.13°, 35.56°, 47.03°, 56.01°, 62.12°, 65.70°, 67.29°, 68.28°, 72.03°, and 76.18° were observed corresponding to [100], [2], [102], [110], [103], [200], [112], [201], [4], and [202] planes for a typical wurtzite polycrystal struture (JCPDS card No 5–0664) [38, 42]. The XRD pattern thus clearly confirmed that the ZnO NPs synthesized by the egg albumin were hexagonal in shape and crystalline in nature. Average size of the ZnO was calculated from the width of the reflection according to the Debye-Scherrer equation: D = (0.9 l/ λ)/ (β cos Ө), where β is the full-width at half maximum (FWHM) of the peak in radians, θ is the angle of diffraction and λ is the wavelength of the X-ray radiation. By considering the FWHM of the most intense diffraction peak (101) of ZnO NPs, the crystalline size of the ZnO NPs was calculated as ~16.7 nm. The XRD data clearly showed that the ZnO NPs in this study has been successfully synthesized by using the egg albumin and it has good purity and crystalline nature [37, 40].

**Fig 2:**
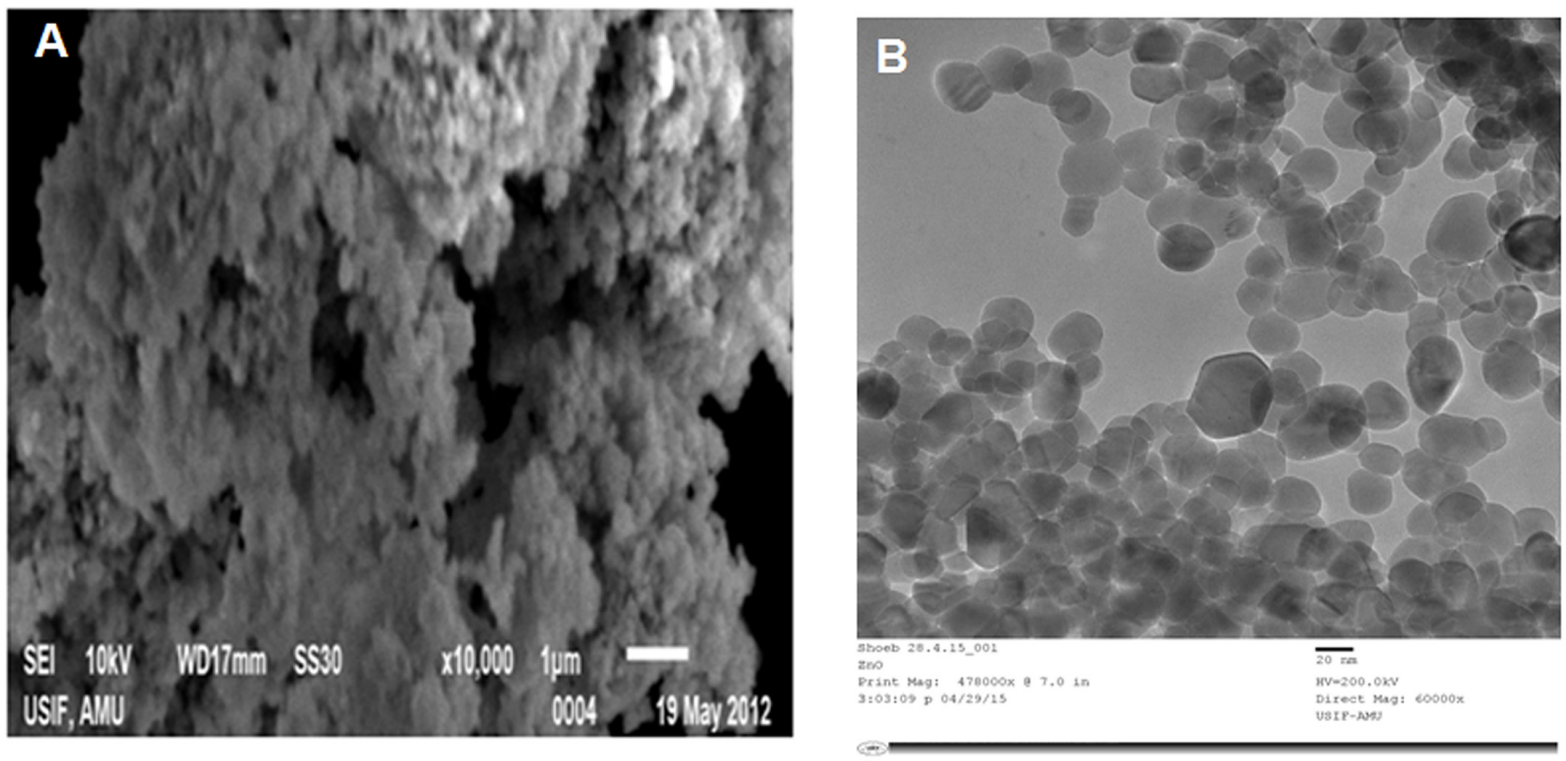
(A) SEM image of ZnO NPs size shows the structure and size distribution varies ZnO NPs depicts clearly the aggregation of ZnO NPs. (B) TEM image shows the structure and size distribution of ZnO NPs.

**Fig 3:**
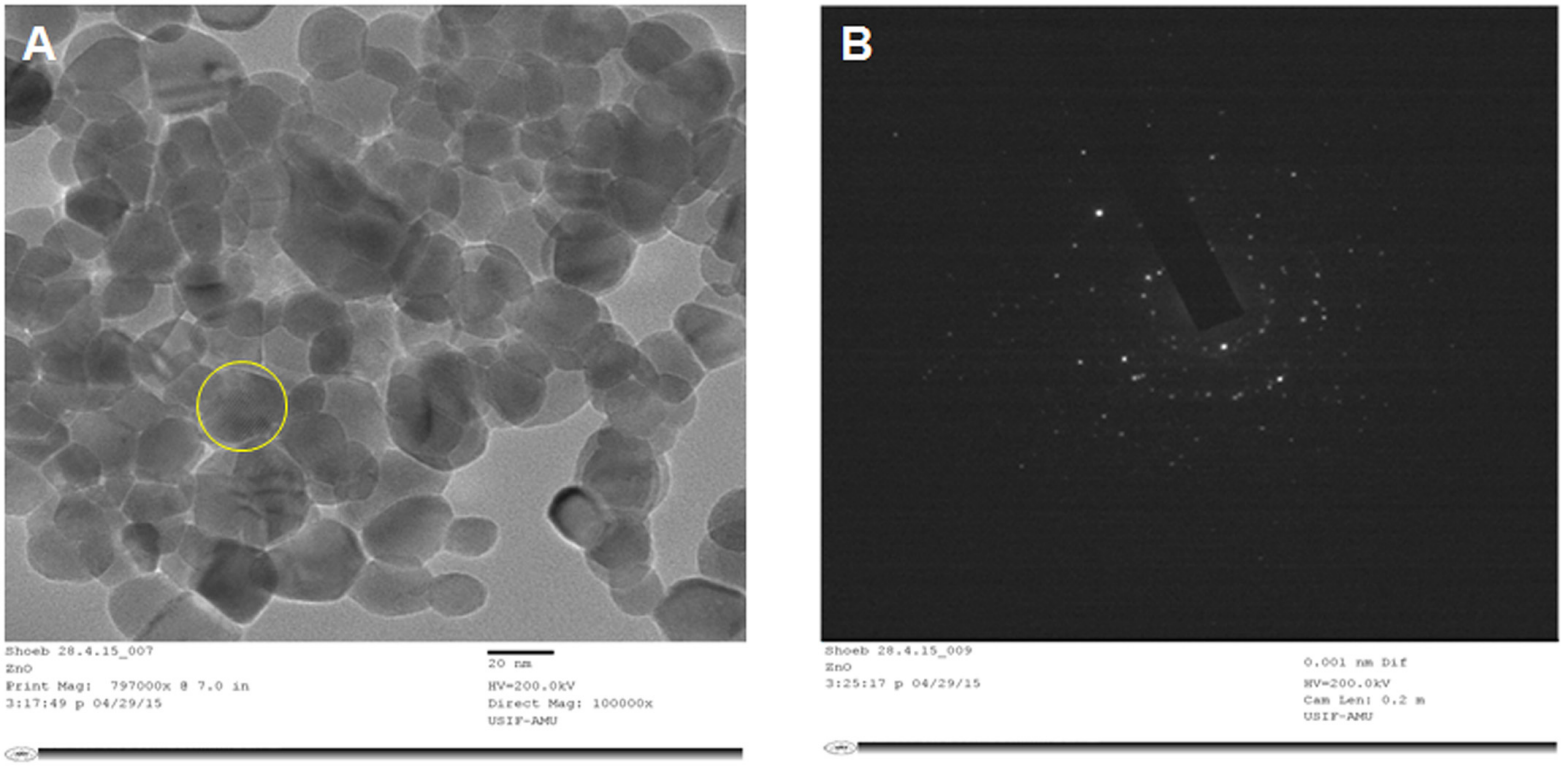
(A) HRTEM image of ZnO NPs. (B) SAED pattern of ZnO NPs.

Fig 2A shows the SEM images of synthesized ZnO NPs (after post-annealing at 400°C in air for 3 h). SEM image clearly indicate that ZnO primary single nanocrystals fused with adjoining crystals and forms secondary ZnO NPs (micrometre clusters). In order to determine the ZnO NPs size and crystalline structure, TEM and SAED techniques were used. The representative TEM images of ZnO NPs are shown in Fig 2B. The analysis shown that the ZnO NPs size distribution varies from ~12 to 50 nm with an average particle size of ~17 nm, which was in good agreement with the particle sizes (~16.7 nm) calculated from the XRD data using Debye-Scherrer equation. The HRTEM images of ZnO NPs and the corresponding SAED were shown in Fig 3A and 3B, respectively. The HRTEM image analysis confirmed that the synthesized ZnO NPs have randomly oriented polycrystalline nature and lattices clearly indicating its nanoscale crystallinity (Fig 3A). The SAED spectrum indicates the polycrystalline nature of ZnO NPs, which indicates that the secondary ZnO NPs are polycrystalline, consisting of much smaller primary crystals of the same crystal orientation (Fig 3B). It may be possible that the egg albumin may behave as a capping agent, causing the individual ZnO primary single nanocrystal to grow up separately and finally assemble to form secondary ZnO NPs via the driven force of van der Waals interaction.

**Fig 4:**
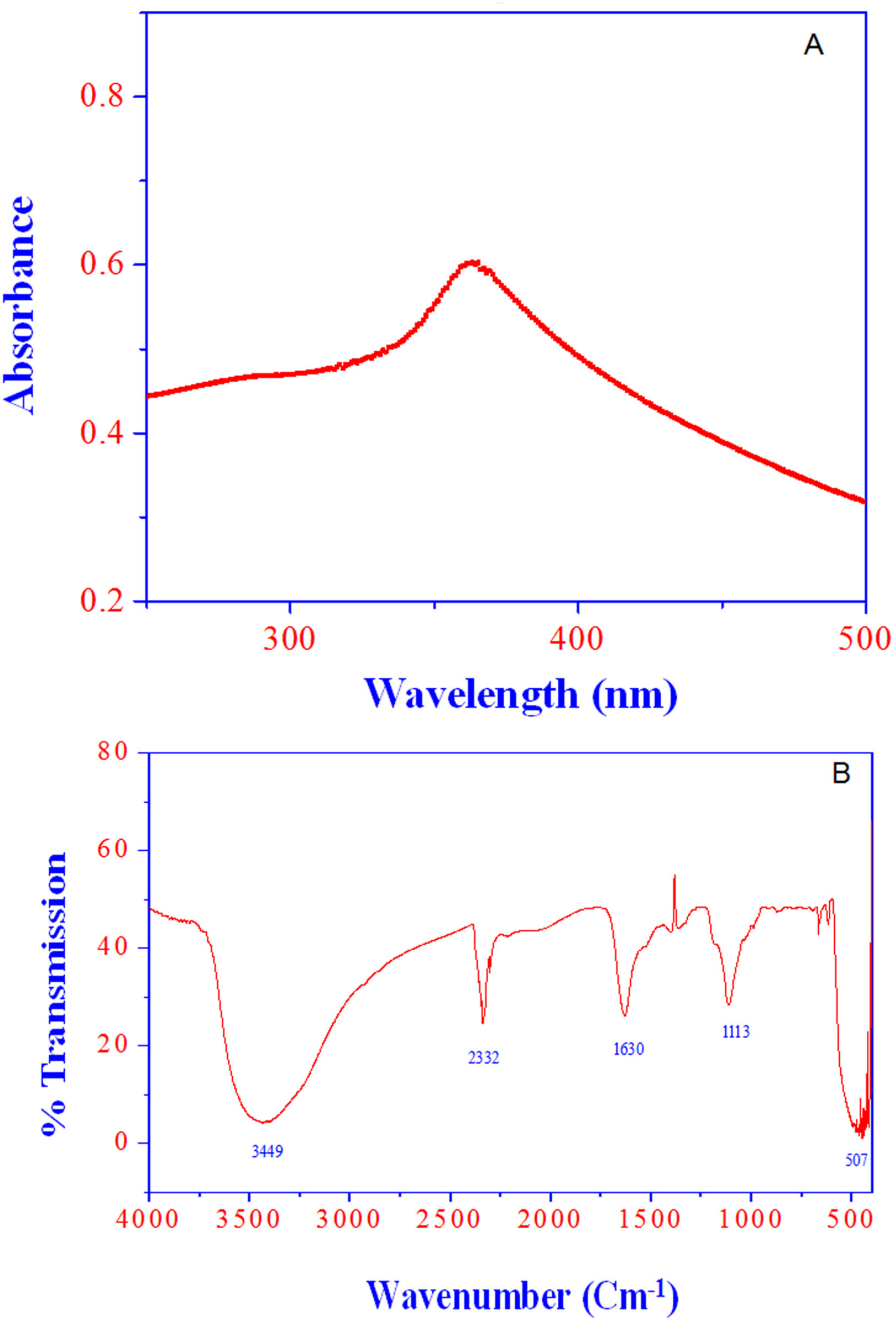
(A) characteristic absorption peak of ZnO NPs (15 μg/mL) was recorded at ~A360 nm wavelength. (B) FTIR spectrum of ZnO NPs.

Fig 4A shows the UV–vis absorption spectrum of the synthesized ZnO NPs. The ZnO NPs sample has an absorption peak at ~362 nm, attributed to the electron (e-) transitions from the valence band (Ev) to the conduction band (Ec) (O_2p_ → Zn_3d_) [37, 47, 48] bond. The optical data indicates that the egg albumin has been successfully removed by the sintering and does not influence the absorption behavior of the ZnO NPs. Fourier transform infrared (FTIR) spectrum of ZnO NPs revealed that the signature vibration band at 507 cm^-1^, which corresponds to E_2_ mode of hexagonal ZnO wurtzite structure [37, 47, 49]. The band at the position 507 cm^-1^ depicts the ZnO NPs stretching frequency of Zn–O elemental bonds (Fig 4B). The intermediate product hydrozincite impurity was not observed in the synthesized ZnO NPs sample because of the absence of the vibration band at 677 cm^-1^ (hydroxide phase). Therefore the, sintering at 400^°^C was able to remove the egg albumen biotemplate from the ZnO NPs. The vibration bands at ~1630 and 3449 cm^-1^ were due to the stretching frequency of hydroxyl groups of absorbed water from the ambient atmosphere [50, 51].

### Anti Helminthic Effects of ZnO NPs

#### Parasite mobility

The parasite mobility was visually monitored after every two hours up to 24 hours and it was observed that motility of the worms was seriously affected with the increasing time of incubation of parasites with ZnO NPs in a concentration dependent manner. Changes in parasite mobility are given in Table 1. The untreated control worms remained active throughout the incubation period.

**Table 1:** The mobility of *Gigantocotyle explanatum* was differentially affected with respect to thetime of their *in vitro* treatment with biocompatible zinc oxide nano particles (ZnO NPs).

Treatments With ZnO NPs	2 Hours	4 Hours	6 Hours	8 Hours	10 Hours	12 Hours	24 Hours
Control	++++	++++	++++	++++	++++	++++	+++
0.004%	++++	++++	++++	++++	++++	+++	++
0.008%	++++	++++	++++	++++	++++	+++	++
0.012%	++++	++++	++++	++++	+++	+++	+

++++(High),

+++ (Moderate),

++(Low),

+(Very Low/negligible).

#### Super Oxide Dismutase (SOD) activity

**Fig 5:**
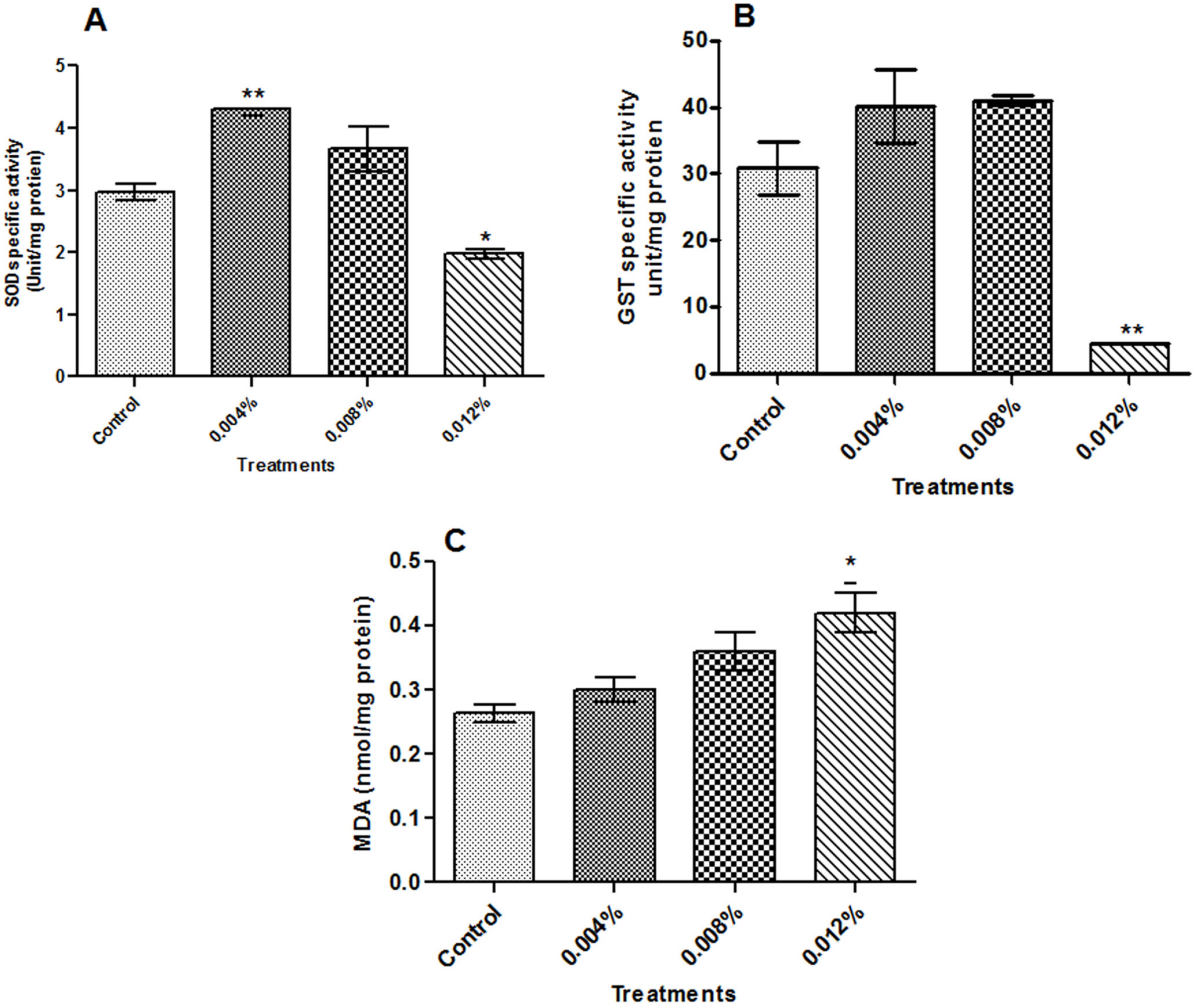
Status of oxidative stress markers: Superoxide dismutase (SOD) (A) and Glutathione-S-transferase (GST) (B) specific activities and malondialdehyde (MDA) (C) level in *Gigantocotyle explanatum*. The data is represented as Mean±SE and (*) p<0.05 and (**), p<0.0.01, was considered as significant.

The incubation of worms with different concentrations of ZnO NPs (0.004%, 0.008% & 0.012%) exert variable effects on the SOD activity. The results are summarized in Table 2 and Fig 5B. The specific SOD activity was higher than the controls at lower concentrations (0.004% (p< 0.01) & 0.008%) of the NPs, indicating an initial stimulatory response after that the enzyme activity dropped significantly (p< 0.05) leading to 34.3 percent inhibition of SOD activity in the worms treated with 0.012% ZnO NPs. (Table 2 and Fig 5A).

**Table 2:** The effect of biocompatible zinc oxide nano particles (ZnO NPs) on the Glutathione-S-Transferase (GST) and Superoxide dismutase (SOD) in *Gigantocotyle explanatum*.

Experimental Setups	Mean specific activity of SOD*	Percent inhibition of SOD	Mean specific activity of GST^†^	Percent inhibition of GST
Control	3.0± 0.13	----	30.9±3.98	----
0.004%	4.3±0.00	Nil	42.1 ±5.51	Nil
0.008%	3.7± 0.37	Nil	41.1 ±.763	Nil
0.012%	1.97±0.07	34.3%	4.5 ± 0.065	85.4%

*Superoxide dismutase: specific enzyme activity in Units/ mg protein.

^†^glutathione-S- transferase: specific enzyme activity in Units/ mg protein.

#### Glutathione-S-transferase (GST) activity

The detoxifying enzyme, Glutathione S- transferase (GST) activity in somatic homogenate of *G*. *explanatum* was not affected by ZnO NPs in a concentration dependent manner. However, the highly significant (p<0.01) inhibition of GST activity was also recorded at the highest concentration (0.012%) of ZnO NPs, while at lower concentrations (0.004% & 0.008%) of ZnO NPs, an insignificant increase of GST activity was observed, thus showing transition phase from active antioxidant defense mechanism (ADM) to exhausted ADM (Fig 2B). However, the treatment of worms with 0.012% concentration of ZnO NPs resulted in a loss of 85% GST activity (Table 2 and Fig 5B).

#### ZnO NPs induces lipid peroxidation process in *G*. *explanatum*

Malondialdehyde (MDA) is an end product of lipid peroxidation process. The level of MDA was significantly increased (p<0.05) at the highest concentration (0.012%) of ZnO NPs while no significant difference was observed at lower concentrations (Fig 5C).

#### Low intensity of polypeptide bands might be due to inhibition / under expressionof the proteins

**Fig 6:**
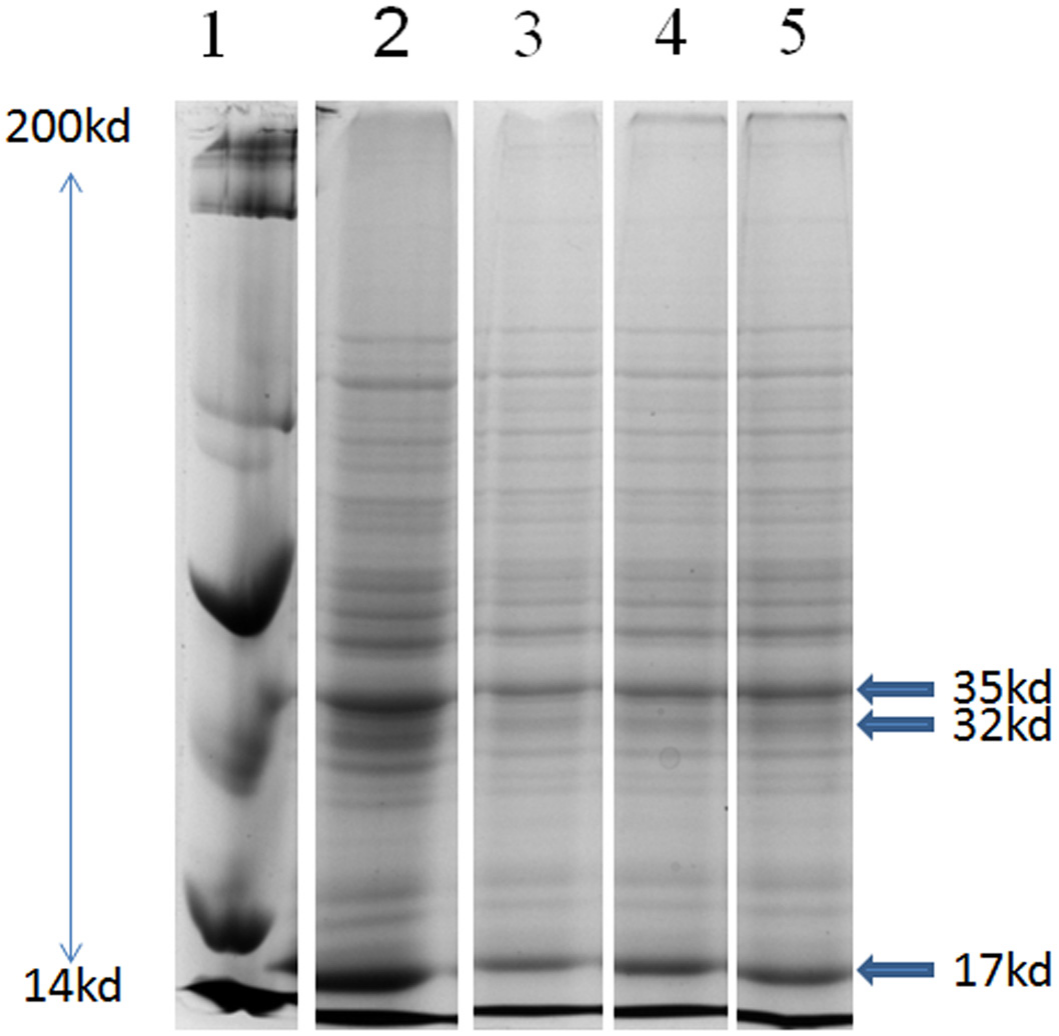
Change in polypeptide profile expression: The polypeptide profile of TCA/Acetone (10%) precipitated somatic proteins following SDS-PAGE. Lane 1: Bio-Rad standard marker, lane: 2 (Control), lanes: 3, 4 & 5 are the protein profiles of the worms treated with 0.004%, 0.008% & 0.012% ZnO NPs, respectively.

The SDS-PAGE profile of TCA/Acetone precipitated *G*. *explanatum* somatic proteins exhibit low intensity bands of apparent molecular weight (M_r_) 32, 35, and 17 kD in all the ZnO NPs treated worms as compared to the untreated controls. This low intensity bands may be the outcome of either degradation, inhibition or under expression of these proteins in response to the treatment with ZnO NPs (Fig 6).

#### Scanning Electron Microscopy (SEM)

**Fig 7:**
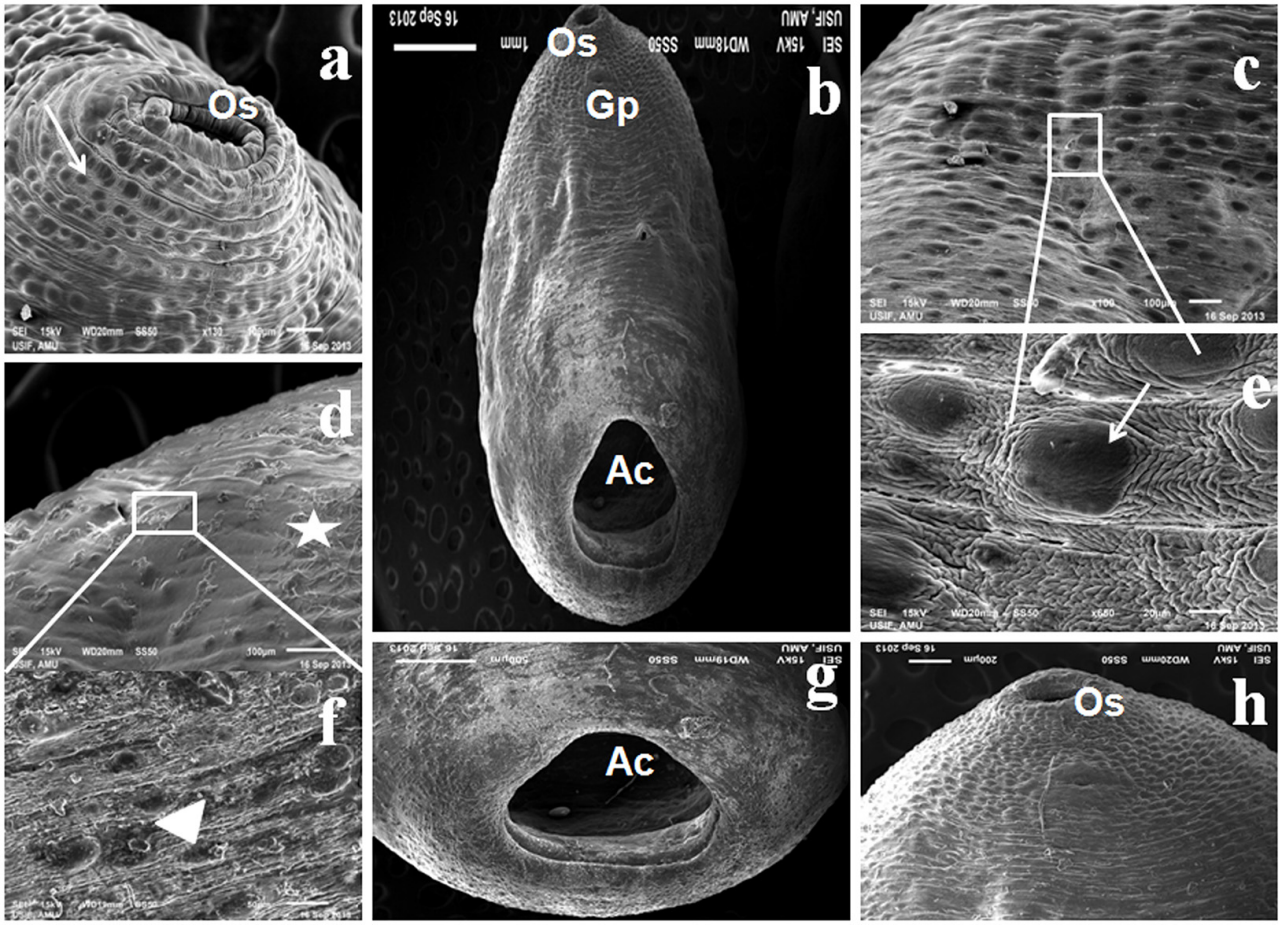
Change in surface topography: Scanning electron micrograph of *Gigantocotyle explanatum* showing the disruption of tegument(star) and surface papillae (arrow head) particularly in the posterior and the acetabular (Ac) region following treatment of worms with 0.012% ZnO NPs (b, d, f, g & h), and the untreated controls (a, c & e) with normal surface topography. An arrow showing the normal papillae. Gp: gonopore; Os: oral sucker.

The results of scanning electron microscopy of *G*. *Explanatum* revealed significant tegumental damage in worms treated with 0.012% ZnO NPs as compared to the untreated controls. Weathering of the tegument resulted in the erosion of surface papillae as well as disruption of surface annulations. These effects were more prominent in the posterior region of the worms, particularly around the acetabulum (Fig 7). It was interesting to note that the oral sucker and the tegumental architecture around it appeared to be unaffected.

#### Histopathology

**Fig 8:**
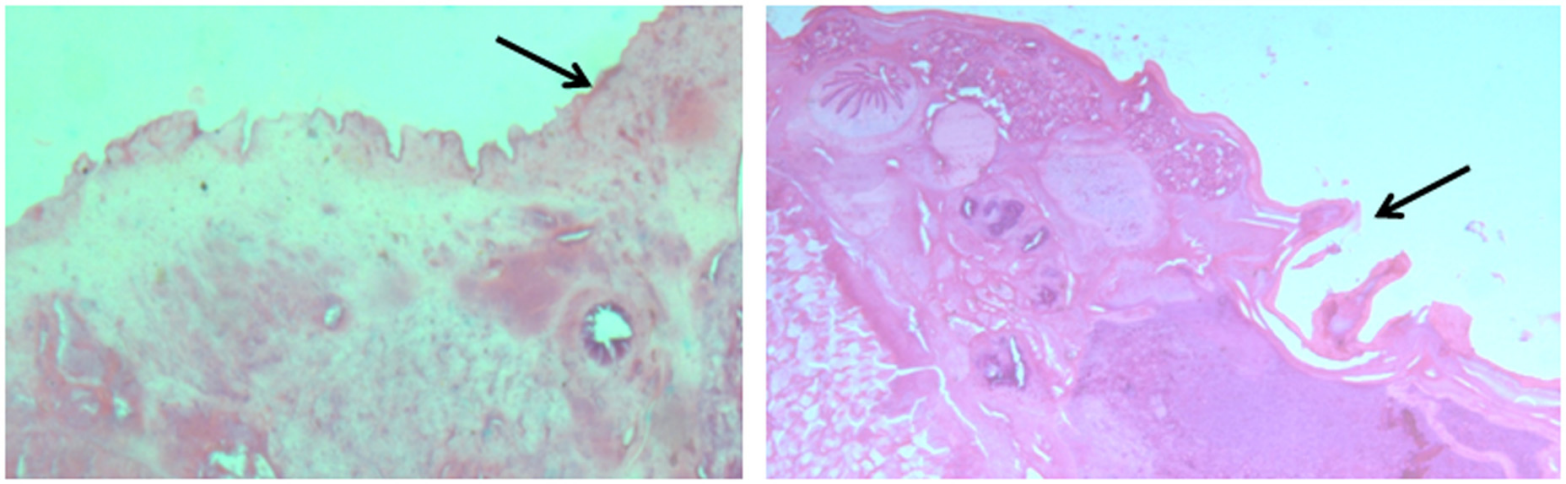
Histological examinations: Longitudinal section of *Gigantocotyle explanatum* at magnification 100x: (Control) showing normal tegumental surface (arrow) and (Treated) the disrupted tegument (arrow).

Profound differences were observed in the histology of *G*. *explanatum* treated with 0.012% O NPs when compared with the untreated control worms. The tegument appeared to be severely damaged with the loss of normal architecture, often exposing the parenchyma below, particularly in the posterior region of the NPs treated worms, whereas no such changes were evident in the control worms incubated without ZnO NPs (Fig 8).

## Discussion

The development of anthelmintic resistance affects the success of treatment in humans and also threatens the productivity of the livestock [5, 38]. The rapid development of resistance following extensive use of anthelmintics in livestock industry has necessitated the development of alternative strategies. The metal derived Zinc Oxide (ZnO) nano particles (NPs) which exhibit strong tendency to generate reactive oxygen species (ROS) in pathogens and develop oxidative stress, have been widely used as antibacterial agent to treat the resistant strains [52, 53]. TiO_2_ and Ag_2_O NPs have also been used as antiproliferative agents against the *Leishmania* parasites [9]. ZnO NPs also induce generation of hydroxyl ions and ROS, which cause membrane damage [53, 54] by electrostatic binding. However, no such information is available on the effect of ZnO NPs on helminths in general and amphistomes in particular. Therefore, present study is the first attempt to investigate anthelmintic effect of biocompatible ZnO NPs (Zno NPs) on a helminth parasite which infects Indian livestock causing substantial economic losses. It has been previously investigated that ZnO NPs are involved in development of oxidative stress that gives anticandidal potency to these NPs [37].

*In vitro* treatment of *Gigantocotyle explanatum* with different concentrations of ZnO NPs produced differential effects. The use of lower concentrations of ZnO NPs (80μg /ml or 0.004% w/v; and 160μg /ml or 0.008% w/v) appeared to have produced oxidative stress due to the production of ROS in the parasites. As a result of which the flukes mount a survival effort by increasing the activity of antioxidant enzymes, SOD and GST, to scavenge the ROS, which might have been produced in response to ZnO NPs treatment since it is well known that the level of intracellular ROS rises in response to the use of nano particles [55]. The SOD catalyzes dismutation of O^2-^ to H_2_O_2_, while GST mediates reaction of electrophilic metabolites of xenobiotics to the GSH (reduced glutathione) [56–58]. Both these enzymes along with other antioxidants form an effective system against ROS. But this protective system appeared to be disrupted when the worms were treated with the highest concentration (240 μg /ml, or 0.012% w/v) of the ZnO NPs in the present study (Fig 5A & 5B). A significant level of inhibition of the GST and SOD activity was recorded in *G*. *explanatum* following treatment of worms with the highest concentration (240 μg /ml, or 0.012% w/v) of the ZnO NPs, possibly due to the saturation of enzymes as a result of over production of hydroxyl ions and ROS which render the detoxification mechanism in *G*. *explanatum* ineffective (Table 2). The oxidative stress generated by NPs, also targets the lipids and initiate the process of lipid peroxidation [59]. The MDA level at the highest concentration (240 μg /ml, or 0.012% w/v) further suggests that the NPs generated enough oxidative stress to lead the process of lipid peroxidation (Fig 5C). The elevated intracellular ROS level could not only affect the potential and permeability of the cell membrane, but also of mitochondria, which are the main cell organelles adversely affected by nano particles [60]. Such changes disrupt the electron transport system ultimately inhibiting ATP production [5], and hence affecting the contractile movement of the parasite (Table 1). The worm motility has been used earlier to test the efficacy of drugs and inhibitors on these worms [61].

Under normal conditions, ROS remain in homeostasis but due to disease, drugs and stress, the ROS level is elevated. The macromolecules like DNA, proteins and lipids are the important targets of ROS [62–64]. The SDS-PAGE results revealed an overall under expression of 35.0 KD, 32.0 KD and 17. KD protein bands in the ZnO NPs treated worms, which might be due to the cellular leakage or weathering of the tegumental surface, however, further studies are necessary to ascertain these observations. The histology and the surface topography of the ZnO NPs treated worms clearly shows pronounced damage in the tegumental papillae and the surface annulations as revealed by scanning electron microscopy, particularly in the posterior region and around acetabulum. The tegumental disruptions would facilitate the penetration of nano particles in the sub-tegumental regions where they can produce deeper lesions in the worms. Since NPs function as soft acids, they have a great affinity for sulphur and phosphorus containing soft bases [55, 65]. Therefore sulphur containing proteins of cell membrane could be the preferred targets of ZnO NPs (240 μg /ml) used in this study.

**Fig 9:**
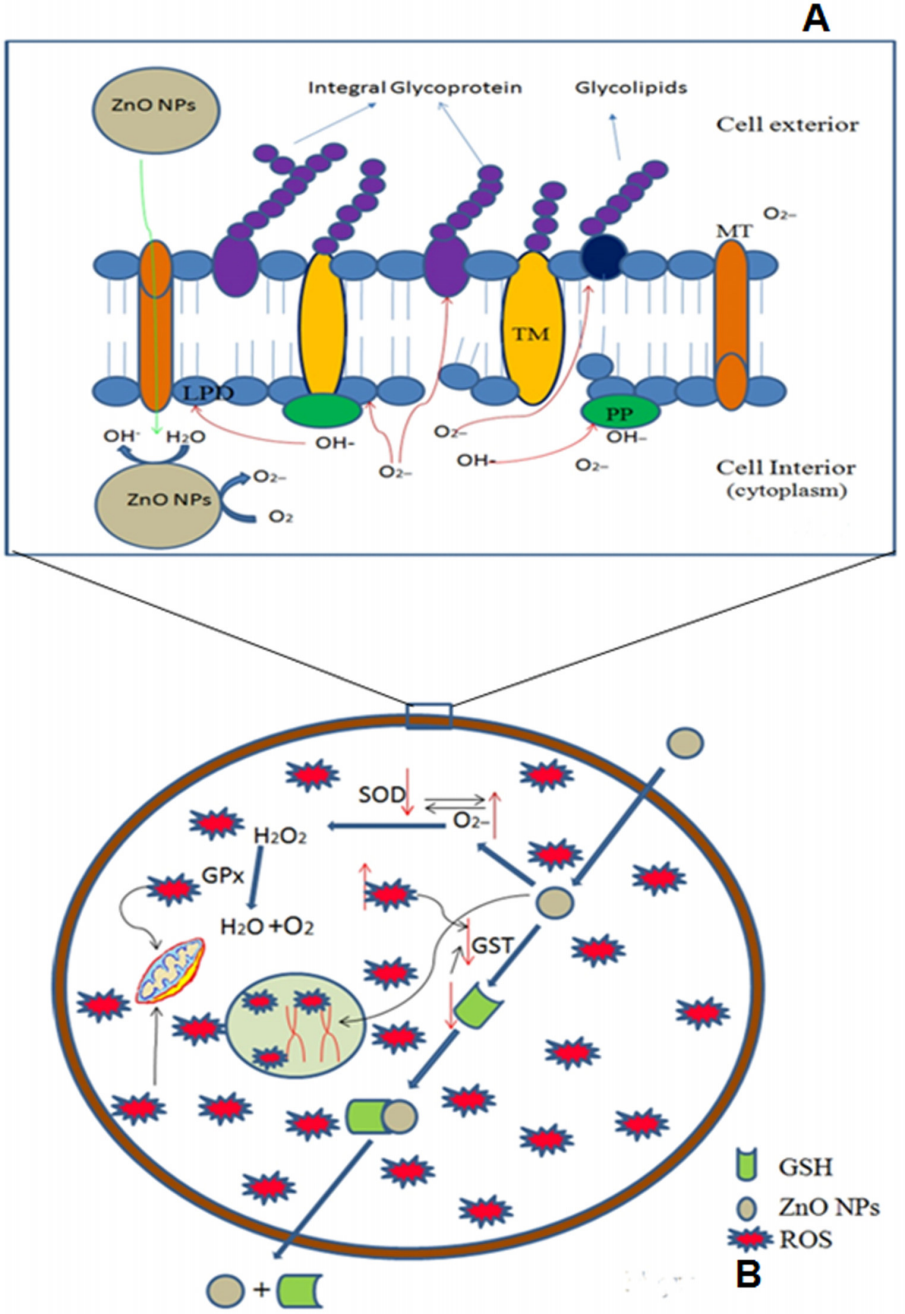
**(A)** Damage of the tegumental surface membrane by ZnO NPs will pave the way for the entry of nanoparticles into the cells through membrane transporters (MT). At the cytoplasmic side ZnO NPs generate ROS in the form of singlet oxygen species and hydroxyl ions. These ROS act upon lipids (LPD) and proteins, trans membrane protein; TM, peripheral proteins; PP, glycolipids and integral glycoproteins of cell membrane and cause damage. **(B)**. The ZnO NPs enter the cells, possibly via the transporters present in the lipid bilayer, and develop oxidative stress resulting into production of ROS, which could stimulate detoxification involving SOD and glutathione peroxidase (GPx). GST activates the conjugation of ZnO NPs with its substrate GSH and exports them outside the cell. But the cellular defense mechanism might be disrupted via increased production of ROS when a high influx of ZnO NPs occurs at the highest concentration of treatment. The increased ROS level might be the causative agent for SOD and GST depletion. Inhibition of these enzymes might be due to the direct action of ROS on these enzymes or depletion of GSH in case of GST. Alternatively ZnO NPs also enter inside the nucleus and damage DNA either by direct attack by Zn+ ions (formed by dissolution of ZnO NPs) or by generation of ROS.

Taken together, our finding confirms a significant inhibition of SOD and GST activity, elevation of MDA, inhibition/ degradation of proteins, loss of motility and disruption of tegument due to treatment of *G*. *explanatum* with 240μg/ml ZnO NPs, thus suggesting strong anthelmintic activity of nanoparticles. In case of *Gigantocotyle explanatum* nothing is known about the mechanism of action of any nano particle, but the different parameters used in the present study could give a plausible mechanism of action of Zinc Oxide nano particles in the parasite under study (Fig 9A and 9B). However, further studies are needed to elucidate the exact mechanism of mode of action and development of *in vivo* treatment strategies of biocompatible ZnO NPs in these and related.
